# Epidemiological analysis of six common respiratory pathogen infections in children in Chengdu from 2022 to 2024

**DOI:** 10.3389/fped.2026.1762543

**Published:** 2026-04-29

**Authors:** Chunyuan Wang, Mengjun Luo, Shuzhe Yang

**Affiliations:** Clinical Laboratory Department, Chengdu Womens and Childrens Central Hospital, School of Medicine, University of Electronic Science and Technology of China, Chengdu, China

**Keywords:** adenoviruses, human rhinovirus, influenza virus, mycoplasma pneumoniae, respiratory pathogens, respiratory syncytial virus, respiratory tract infection

## Abstract

**Background:**

This study aimed to describe the epidemiological characteristics of six common respiratory pathogens in children admitted to Chengdu Women's and Children's Central Hospital.

**Methods:**

A total of children with respiratory tract infection symptoms, including fever, cough, and headache, who were admitted to the hospital from September 2022 to December 2024 were enrolled in this study. Multiplex reverse transcription quantitative polymerase chain reaction (RT-qPCR) was used to detect the six respiratory pathogens and analyze their epidemiological characteristics.

**Results:**

Among the 64,500 included children, 34,505 (53.50%) tested positive for respiratory pathogens. Human rhinovirus (HRV) had the highest positive rate of 18.67% (12,042/64,500), followed by *Mycoplasma pneumoniae* (MP) with a positive rate of 14.93% (9,632/64,500). The study population consisted of 36,020 males and 28,480 females, with positive rates of 53.91% (19,417/36,020) and 52.98% (15,088/28,480), respectively. The overall positive rate showed an increasing trend with age: the highest positive rate (63.61%) was found in children aged ≥6 years, while the lowest (37.01%) was observed in infants aged <1 year. Significant seasonal variations were noted in the detection rates of the six pathogens, with the highest rate in winter and the lowest in summer. Pneumonia was the most common clinical manifestation of respiratory infections associated with these pathogens.

**Conclusions:**

In the post-epidemic era, children remain susceptible to infection by various respiratory pathogens. Therefore, continuous epidemiological analysis and activity surveillance of respiratory pathogens can provide a scientific basis for early clinical diagnosis, promotion of rational drug use, and development of effective prevention and control strategies.

## 1 Introduction

Respiratory tract infection (RTI) is an infectious disease affecting the respiratory system. It is caused by pathogens such as bacteria, viruses, mycoplasmas, and fungi that invade the human respiratory tract, including the nasal cavity, pharynx, trachea, bronchi, and lungs. Key pathogens, such as respiratory syncytial virus (RSV), influenza virus (IFV), adenoviruses (ADV), and *Mycoplasma pneumoniae* (MP), are characterized by high infectivity and rapid transmission, posing a significant threat to public health (1, 2).

RSV is a well-documented cause of acute lower respiratory tract infection (ALRTI) in infants and young children (3). Nearly all children experience their first RSV infection by the age of two (4). Because of incomplete immunity after primary infection, reinfection is common (5, 6). Influenza virus (IFV) causes acute upper respiratory tract infections and spreads rapidly via airborne transmission, leading to seasonal epidemics and occasional global pandemics (7, 8). Adenoviruses (ADV), belonging to the family Adenoviridae, can infect the respiratory tract, eyes, kidneys, gastrointestinal tract, and blood cells, with high prevalence reported in certain regions of China (9). *Mycoplasma pneumoniae* (MP), a prokaryote lacking a cell wall, is a major etiological agent of community-acquired pneumonia (CAP) in children aged 5 years and older, accounting for approximately 10% to 40% of pediatric CAP cases (10). Additionally, human rhinovirus (HRV), a member of the family Picornaviridae, can not only cause upper respiratory tract infections but also induce lower respiratory tract diseases such as bronchitis, bronchopneumonia, and acute asthma in infants and children.

Public health interventions, including lockdowns, movement restrictions, mask mandates, and school closures (11) were implemented during the SARS-CoV-2 pandemic to break infection chains by modifying key human behaviors. Consequently, the epidemiology of other respiratory pathogens was substantially affected (12, 13). A study from the USA showed a 98% decrease in positive influenza tests among adults and children during the lockdown periods (14). However, following the easing of these restrictions in China at the end of 2022, a substantial increase in pediatric respiratory tract infection cases was observed in 2023 and 2024. Despite this, the primary pathogens driving these infections remain understudied. Furthermore, previous studies on the epidemiological characteristics of respiratory pathogens are often limited to one or a few clinical settings, covering only a small subset of the infected population (15, 16). For example, many studies have focused solely on community-acquired pneumonia (CAP), excluding upper respiratory tract infections, making it difficult to assess the true public health impact of these pathogens. This study was conducted to analyze infections with six pathogens in pediatric inpatients and outpatients with respiratory infections during the final phase of public health interventions and after their discontinuation. It aims to quantify the etiological spectrum and dynamic evolution of these pathogens among children in the post-pandemic era, assess clinical characteristics in the new epidemic seasons, and generate critical data to inform future children's health strategies.

## 2 Materials and methods

### 2.1 Patients and groups

From September 1st, 2022 to December 31st, 2024, a total of 64,500 children diagnosed with acute upper respiratory tract infection, pneumonia, bronchitis, etc. were enrolled in this study (see Table 1 for details). Inclusion criteria: (1) Pneumonia: The children (≤18 years old) were required to meet two or more of the following clinical features: Clinical Symptoms: New onset or worsening of cough, sputum production, fever, tachypnea, grunting, or cyanosis. Signs: Presence of fixed moist rales and/or signs of pulmonary consolidation in lung auscultation; may be accompanied by nasal flaring and/or chest wall retractions. Chest Imaging: Newly identified patchy infiltrates, lobar or segmental consolidation, ground-glass opacities, or interstitial changes, with or without pleural effusion. Pediatric patients with pneumonia who meet any one of the following criteria are diagnosed as severe pneumonia: the presence of chest wall indrawing on inspiration, nasal flaring, or grunting, which indicates hypoxemia, is defined as severe pneumonia; or the presence of central cyanosis, severe respiratory distress, refusal to feed or dehydration, or disturbed consciousness (such as lethargy, coma, convulsions) (17). (2) Acute Upper Respiratory Tract Infection (AURI): The children (≤18 years old) were required to meet two or more of the following clinical features: Local Symptoms: Rhinorrhea, nasal congestion, sneezing, sore throat, cough. Systemic Symptoms: Fever (axillary temperature  ≥ 37.5 °C), chills, headache, malaise, loss of appetite. Clinical Signs: Pharyngeal congestion, redness and swelling of the tonsils, significant enlargement of submandibular lymph nodes (18). (3) Bronchitis: Inflammation of the bronchi; respiratory symptoms such as cough, sputum production, chest tightness, and fever; some patients may also experience shortness of breath and chest pain. Physical examination may reveal dry or wet rales in the lungs during auscultation (18). (4) Children with suspected respiratory infection and minimal symptoms.

**Table 1:** General characteristics of the patients.

Characteristic	Category	Median [IQR]
Age (years）	Overall	3 [1,5]
	<1-year (month)	3 [1,7]
	1−2 years(year)	1 [1,2]
	3−5 years(year)	4 [3,4]
	≥6 years (year)	7 [6,9]

Age was presented as median [interquartile range, IQR]; for children under 1 year old, age was recorded in months, while for children over 1 year old, age was recorded in integer years (e.g., children aged 1 year and several months were automatically recorded as 1 year).

Exclusion Criteria: (1) Age ≥18 years; (2) Lack of a clear clinical diagnosis or incomplete clinical data.

The study was approved by the Research Ethics Commission of Chengdu Women's and Children's Central Hospital [Lot No.: 2025(67)]. The access to patient records was authorized, and all patients’ information was kept confidential.

### 2.2 Sample collection

All respiratory specimens were collected at the first visit or within 3 days of admission for inpatients. Bilateral swabs of the pharyngeal tonsils and posterior pharyngeal walls were obtained using sterile throat swabs. The swabs were placed in a sterile tube containing preservation solution and send for testing as soon as possible. The specimens are stable for 48 h at 4 °C, 10 months at −20 °C, and 12 months at −70 °C, and should not undergo more than four freeze-thaw cycles.

### 2.3 Pathogen detection

Respiratory pathogen nucleic acids were detected by multiplex RT-qPCR (Six Nucleic Acid Detection Kits for respiratory pathogens, Sheng xiang Biotechnology). Nucleic acid was extracted from 300 μL of each test sample, as well as the negative and positive controls, using a nucleic acid extraction kit (Shengxiang Biotechnology) on the Nucleic Acid Extraction System (Shengxiang Biotechnology). Subsequently, 5 μL of the extracted nucleic acid from each sample and control (negative and positive controls) was aliquoted into individual 0.2 mL PCR tubes. Each aliquot was mixed with 45 μL of PCR master mix. The PCR amplification was performed under the following conditions: reverse transcription at 50 °C for 30 min and initial denaturation at 95 °C for 1 min; followed by 45 cycles of denaturation at 95 °C for 15 s and annealing/extension at 60 °C for 60 s; with a final hold at 25 °C for 10 s The positive result was defined an obvious S-type amplification curve with a Ct value ≤40. A negative result was definedas no amplification curve or Ct value >40. Quality control:The PCR detection system incorporates a positive internal control, by which the normal amplification of the human housekeeping gene encoding glyceraldehyde-3-phosphate dehydrogenase (GAPDH) in the sample is monitored to oversee the sample extraction and PCR amplification process, thereby avoiding false negative results. To monitor for cross-contamination and false positives, a negative control was included in each run. To ensure the validity of the experimental reagents and conditions, a positive control was used. Only when both controls yielded expected results were the sample data considered valid for analysis

### 2.4 Performance characteristics of the assay

The performance of this kit was validated using in-house reference panels: Detection of 20 positive reference samples yielded a positive coincidence rate of 100%. Detection of 12 negative reference samples yielded a negative coincidence rate of 100%. Repeated testing (10 times) of 11 precision reference samples showed that the coefficient of variation (CV, %) of the Ct values for the corresponding pathogen detection channels was ≤5%. Testing of 20 limit of detection (LoD) reference samples confirmed that all met the required minimum detection limit.

### 2.5 Statistical analysis

Epidemiological, demographic, clinical, laboratory and outcome data were collected using standardized data collection forms. The original data were processed by Excel software. The categorical variables were presented as the number of cases (n) and/or percentages (%). The positive rate for each pathogen was calculated as the number of positive cases for that pathogen divided by the total number of patients tested during the same period. Monthly positive rates were calculated by dividing the number of positive cases in a given month by the total number of patients performed in that month. SPSS 16.0 statistical software (SPSS Inc, Chicago, IL, USA) was used to analyze the data. The chi-square test was used to compare the categorical variable groups. A *P*-value < 0.05 was considered statistically significant.

## 3 Results

### 3.1 Detection of six respiratory pathogens

A total of 64,500 specimens collected from children with respiratory tract infections were tested in this study (inpatient-to-outpatient ratio = 5.76:1). Of these specimens, 34,505 tested positive for at least one pathogen, with an overall positive rate of 53.50%. Among all enrolled children, HRV exhibited the highest positive rate at 18.67% (12,042/64,500), followed by MP at 14.93% (9,632/64,500), ADV at 12.12% (7,816/64,500), RSV at 9.48% (6,114/64,500), FluA at 4.44% (2,863/64,500), and FluB at 1.75% (1,127/64,500). In total, 29,638 children were infected with a single pathogen, 4,649 with two pathogens, 214 with three pathogens, and 4 with four pathogens. The co-infection rate (infection with two or more pathogens) was 7.55% among all enrolled children (see Table 2 for details).

**Table 2:** Single infections and co-infections with different pathogens.

Pathogens	*n* (%)	Pathogens	*n* (%)
HRV	8893 (13.79)	MP + FluB	162 (0.25)
MP	7257 (11.25)	FluA + HRV	152 (0.24)
ADV	5856 (9.08)	FluA + MP	144 (0.22)
RSV	4610 (7.15)	FluA + ADV	129 (0.2)
FLUA	2237 (3.47)	FluA + RSV	116 (0.18)
FLUB	785 (1.22)	FluB + RSV	53 (0.08)
HRV + MP	1168 (1.81)	FluB + ADV	39 (0.06)
HRV + ADV	1032 (1.60)	FluB + HRV	33 (0.05)
HRV + RSV	594 (0.92)	FluB + FluA	27 (0.04)
MP + ADV	389 (0.60)	Three kinds	214 (0.33)
MP + RSV	360 (0.56)	Four kinds	4 (0.01)
RSV + ADV	251(0.39)		

The overall positive cases include single infection and co-infection. *n* denotes the number of positive cases.

% represents the proportion of positive cases among the total tested subjects.

RSV, Respiratory syncytial virus; MP, *Mycoplasma pneumoniae;* FluA, Influenza virus A; FluB, Influenza virus B; Adv, Adenoviruses; HRV, Human rhinovirus.

### 3.2 Pathogen detection results in children of different genders

A total of 36,020 male and 28,480 female children were enrolled in this study. Among male participants, 19,417 tested positive for at least one pathogen, corresponding to a positive rate of 53.91% [95% confidence interval (CI): 53.39–54.42]. In contrast, 15,088 female children tested positive, with a positive rate of 52.98% (95% CI: 52.40–53.56). Statistical analysis revealed a significant difference in the overall positive rate between male and female children (*χ*^2^ = 5.513, *P* = 0.019). With regard to specific pathogens, statistically significant gender-related differences were observed in the positive rates of MP, FluB, and HRV, whereas no significant differences were found in the positive rates of RSV, FluA, and ADV (see Table 3 for details).

**Table 3:** Infection status of six pathogens in children with different sexes.

Sex	male		female			
Pathogen	*n*	%	*n*	%	*X^2^*	*P*
RSV	3467	9.63	2647	9.29	2.030	0.154
MP	5169	14.35	4463	15.67	21.824	0.000
FluA	1596	4.43	1267	4.45	0.012	0.924
FluB	677	1.88	450	1.58	8.308	0.004
ADV	4360	12.10	3456	12.13	0.014	0.906
HRV	7010	19.46	5032	17.67	33.669	0.000

*n* denotes the number of positive cases. % represents the positivity rate (number of positive cases/total tested cases in each subgroup). RSV, Respiratory syncytial virus; MP, *Mycoplasma pneumoniae;* FluA, Influenza virus A; FluB, Influenza virus B; Adv, Adenoviruses; HRV, Human rhinovirus.

### 3.3 Respiratory pathogen infections in different age groups

**Figure 1:**
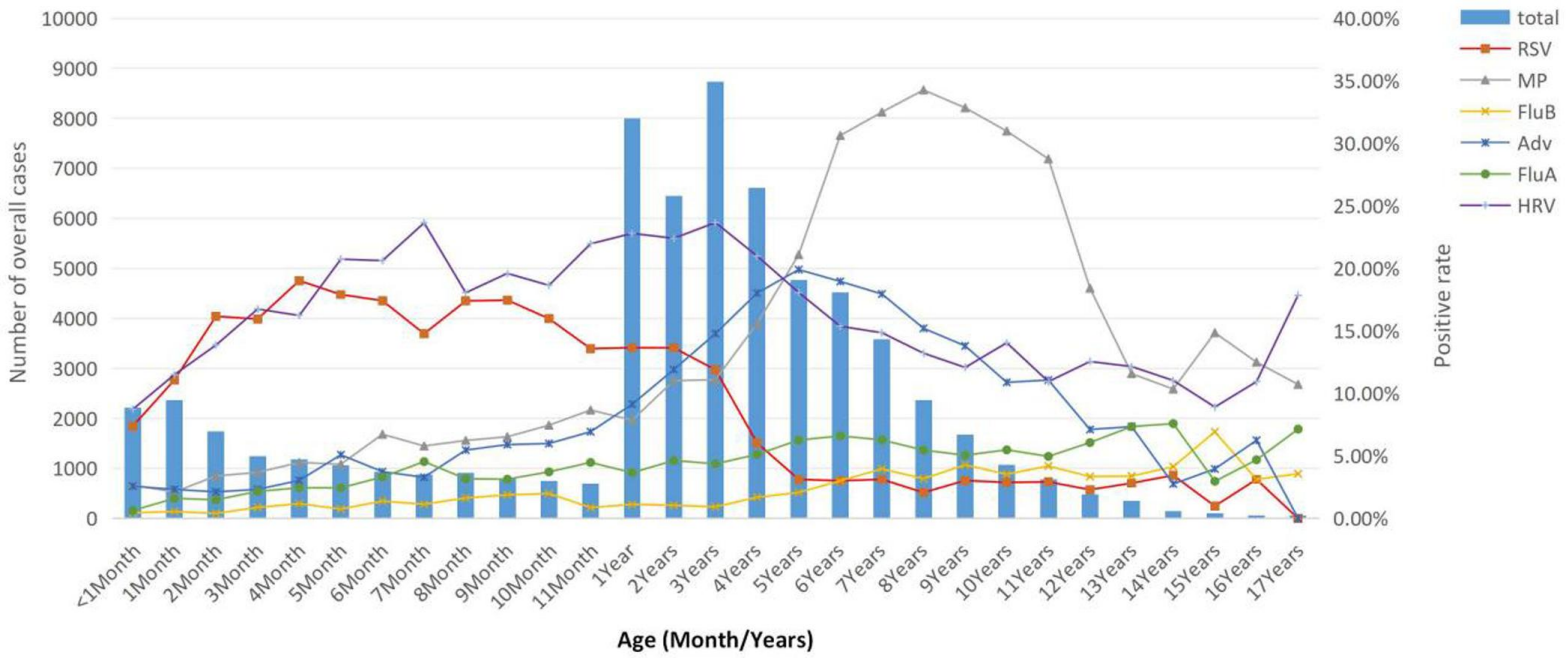
Number of children tested and positive rates of pathogens in different age groups.

Children were divided into four age groups. In the under 1-year group, 14,776 children were tested (inpatient-to-outpatient ratio = 21.7:1), among whom 5,469 were positive, with a positive rate of 37.01% (95% CI: 36.23-37.79). In the 1–2 years group, 14,451 children were tested (inpatient-to-outpatient ratio = 5.3:1), among whom 7,664 were positive, with a positive rate of 53.03% (95% CI: 52.22–53.85). In the 3–5 years group, 20,099 children were tested (inpatient-to-outpatient ratio = 4.9:1), among whom 11,720 were positive, with a positive rate of 58.31% (95% CI: 57.63–58.99). In the ≥6 years group, 15,174 children were tested (inpatient-to-outpatient ratio = 3.8:1), among whom 9,652 were positive, with a positive rate of 63.61% (95% CI: 62.84–64.37). The overall positive rate differed statistically significantly among age groups (*χ*^2^ = 2426.090, *P* < 0.001). The overall positive rate increased with age, as did the positive rates of MP, FluA, and FluB. In contrast, the positive rate of RSV decreased with age. The age-related trends for HRV and ADV were non-linear, showing an initial increase followed by a decrease. The positive rate of HRV was highest in children aged 1–2 years, while ADV peaked in the 3–5-year age group (see Table 4 for details). A finer analysis by age group revealed distinct age-specific patterns. RSV mainly infects young children, with an infection rate exceeding 5% in those aged 0–4 years. In contrast, MP and ADV predominantly infected older children, with infection rates exceeding 10% among children aged over 1 year. MP peaked at 34.27% in the 8-year-old group, whereas ADV reached its peak (19.9%) in the 5-year-old group and then gradually declined. The infection rates of FluA and FluB were relatively low in Chengdu, with FluB showing an even lower rate of less than 5%. HRV maintained a high infection rate across all age groups of children, peaking at 22.66% in the 3-year-old group (Figure 1).

**Table 4:** Positive rates of six pathogens in different age groups.

Age	<1year	1−2years	3−5years	≥6years		
Pathogen	*n* (%)	*n* (%)	*n* (%)	*n* (%)	*X^2^*	*P*
RSV	2125 (14.39)	1972 (13.65)	1586 (7.90)	431 (2.84)	1544.760	0.000
MP	652 (4.41)	1340 (9.27)	3003 (14.94)	4637 (30.56)	4568.415	0.000
FluA	344 (2.33)	592 (4.10)	1018 (5.06)	909 (5.99)	263.891	0.000
FluB	137 (0.93)	158 (1.09)	292 (1.45)	540 (3.56)	394.062	0.000
ADV	522 (3.53)	1499 (10.37)	3429 (17.06)	2366 (15.59)	1697.055	0.000
HRV	2341 (15.84)	3269 (22.62)	4313 (21.46)	2119 (13.96)	550.543	0.000

n denotes the number of positive cases. % represents the positivity rate (number of positive cases/total tested cases in each subgroup). RSV, Respiratory syncytial virus; MP, *Mycoplasma pneumoniae;* FluA, Influenza virus A; FluB, Influenza virus B; Adv, Adenoviruses; HRV, Human rhinovirus.

### 3.4 Seasonality and monthly infection

#### 3.4.1 Seasonal distribution of pathogen positive rates

The overall positive rates of the six pathogens in spring, summer, autumn and winter were 51.78% (95% CI:50.98–52.57), 47.49% (95% CI:46.76–48.23), 56.55% (95% CI:55.81–57.29), 59.09% (95% CI:58.28–59.90), respectively. Among the four seasons, the highest pathogen positive rate was observed in winter, while the lowest was in summer. Statistical analysis revealed significant differences in pathogen positive rates across different seasons (*χ*^2^ = 519.286, *P* < 0.001). Notably, distinct seasonal epidemic patterns were observed among different pathogens. RSV and FluA infections were predominantly prevalent in winter and spring. MP infections mainly peaked in autumn and winter. Although FluB maintained continuous low-level transmission throughout the year, its positive rate increased markedly in winter. ADV was primarily circulating in summer and autumn. HRV maintained high overall activity, with transmission concentrated in autumn and spring (Table 5).

**Table 5:** Positive rates of six pathogens in different seasons.

Pothogen	spring	summer	autumn	winter		
	*n* (%)	*n* (%)	*n* (%)	*n* (%)	*X^2^*	*P*
RSV	2101 (13.85)	347 (1.96)	1239 (7.15)	2427 (17.04)	2611.695	0.000
MP	1443（9.50）	2514 (14.17)	3116 (17.98)	2559 (17.96)	590.134	0.000
FluA	942 (6.21)	238 (1.34)	488 (2.82)	1195 (8.39)	1144.408	0.000
FluB	127 (0.84)	8 (0.05)	161 (0.93)	831 (5.83)	1825.656	0.000
ADV	1150 (7.57)	3265 (18.40)	2192 (12.65)	1209 (8.49)	1132.938	0.000
HRV	3144 (20.71)	2917 (16.44)	4125 (23.8)	1856 (13.03)	699.293	0.000

*n* denotes the number of positive cases. % represents the positivity rate (number of positive cases/total tested cases in each subgroup). RSV, Respiratory syncytial virus; MP, *Mycoplasma pneumoniae;* FluA, Influenza virus A; FluB, Influenza virus B; Adv, Adenoviruses; HRV, Human rhinovirus.

#### 3.4.2 Temporal trend of pathogen positive rates (September 2022–December 2024)

**Figure 2:**
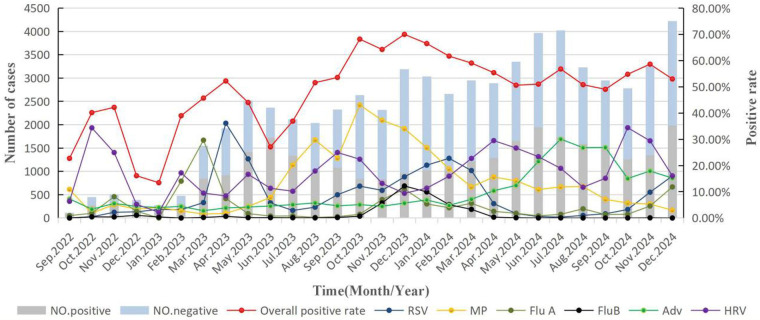
Monthly numbers of positive and negative samples and pathogen positive rates.

We further analyzed the temporal trends of the overall pathogen positive rate and the positive rates of the six individual pathogens from September 2022 to December 2024. The overall positive rate of respiratory pathogens was the lowest in January 2023 (13.46%), followed by a significant increase, with the first peak occurring in March 2023 (52.24%). The highest overall positive rate was recorded in December 2023 (70.01%), after which the rate fluctuated around 50% throughout 2024 (Figure 2). Regarding individual pathogens, the monthly positive rate of RSV increased sharply in April 2023, reaching 36.12% (the first peak), followed by a gradual decline. A second peak was observed in February 2024 (22.75%), with a further increase noted in November 2024, indicating a distinct single annual epidemic peak for RSV. It is noteworthy that the 2023 peak of RSV was delayed until April, which may be attributed to the impact of the COVID-19 pandemic. The positive rate of MP increased significantly from July 2023 (20.21%) and reached its peak in October 2023 (43.09%). However, it gradually decreased from 15.62% to 2.96% between April and December 2024. The infection curve of FluA showed two distinct peaks: the first in March 2023 (29.66%) and the second in December 2023 (11.01%). For FluB, a single infection peak was observed in March 2023 (12.20%), and influenza activity remained generally low in Chengdu thereafter; between April and December 2024, the positive rate of FluB remained below 1%. The positive rate of ADV remained low from September 2022 to March 2024, then increased gradually and peaked in July 2024 (30.08%). HRV was the most frequently detected pathogen among the six, with persistent year-round prevalence.

### 3.5 Disease spectrum associated with different pathogens in children

The most frequent type of respiratory infection attributed to the six pathogens was pneumonia, followed by bronchitis. This pattern was not observed for ADV, which was associated with a higher proportion of acute upper respiratory tract infections than bronchitis. (see Table 6 for details)

**Table 6:** Clinical status of diseases caused by various pathogens in different age groups.

		pneumonia	Severe pneumonia	Upper respiratory tract infections	bronchitis	Others*		
Pathogen	total	*n*(%)	*n*(%)	*n(*%)	*n*(%)	*n*(%)	*X^2^*	*P*
RSV								
<1year	2125	1525 (71.76)	216 (10.16)	27 (1.27)	275 (12.94)	82 (3.86)	4565.394	0.000
1−2year	1972	1348 (68.36)	86 (4.36)	71 (3.6)	339 (17.19)	128 (6.49)	3749.649	0.000
3–5year	1586	1053 (66.39)	35 (2.21)	58 (3.66)	291 (18.35)	149 (9.39)	2826.296	0.000
≥6years	433	202 (46.65)	18 (4.16)	24 (5.54)	88 (20.32)	101 (23.33)	319.734	0.000
MP								
<1year	652	483 (70.08)	36 (5.52)	21 (3.22)	63 (9.66)	49 (7.52)	1498.995	0.000
1–2year	1340	984 (73.43)	38 (2.84)	51 (3.81)	130 (9.7)	137 (10.22)	3026.353	0.000
3–5year	3003	2158 (71.86)	154 (5.13)	95 (3.16)	286 (9.52)	310 (10.32)	6376.955	0.000
≥6years	4636	3162 (68.21)	338 (7.29)	141 (3.04)	442 (9.53)	553 (11.93)	8540.556	0.000
FLuA								
<1year	344	189 (54.94）	19 (5.52)	37 (10.76)	63 (18.31)	36 (10.47)	346.090	0.000
1–2year	592	233 (39.36)	6（1.01）	108 (18.24）	113 (19.09）	132 (22.30)	275.435	0.000
3–5year	1018	377 (37.03)	7 (0.69)	158 (15.52)	256 (25.15)	220 (21.61)	453.175	0.000
≥6years	910	219 (24.07)	9 (0.99)	196 (21.54)	209 (22.97)	277 (30.44)	283.297	0.000
FLuB								
<1year	137	79 (57.66)	6 (4.38)	15 (10.95)	16 (11.68)	21 (15.33)	157.172	0.000
1–2year	158	78 (49.37)	5 (3.16)	12 (7.59)	29 (18.35)	34 (21.52)	128.845	0.000
3–5year	292	119 (40.75)	2 (0.68)	31 (10.62)	57 (19.52)	83 (28.42)	175.753	0.000
≥6years	540	158 (29.26)	7 (1.30)	53 (9.81)	95 (17.59)	227 (42.04)	347.870	0.000
ADV								
<1year	522	333 (63.79)	21 (4.02)	57 (10.92)	53 (10.15)	58 (11.11)	793.285	0.000
1–2year	1499	565 (37.69)	13 (0.87)	297 (19.81)	212 (14.14)	412 (27.48)	720.859	0.000
3–5year	3429	1046 (30.50）	21（0.61）	661 (19.28)	628 (18.31)	1073 (31.29)	1322.51	0.000
≥6years	2365	412 (17.42)	15 (0.63)	591 (24.99）	386 (16.32)	961 (40.63)	1250.322	0.000
HRV								
<1year	2341	1685 (71.98)	108 (4.61)	109 (4.66)	251 (10.72)	188 (8.03)	4979.333	0.000
1–2year	3269	1807 (55.28)	96 (2.94)	289 (8.84)	518 (15.85)	559 (17.1)	3444.323	0.000
3–5year	4313	2375 (55.07)	73 (1.69)	309 (7.16)	775 (17.97)	781(18.1)	4682.975	0.000
≥6years	2119	930(43.89)	77(3.63)	165(7.79)	356(16.8)	591(27.89)	1404.079	0.000

Total represents the number of positive cases for each pathogen in different age groups. *n* denotes the number of positive cases diagnosed with each disease in each group, and % represents the corresponding percentage of different disease diagnoses among these positive cases. RSV, Respiratory syncytial virus; MP, *Mycoplasma pneumoniae;* FluA, Influenza virus A; FluB, Influenza virus B; Adv, Adenoviruses; HRV, Human rhinovirus.

* including fever, headache, allergic rhinitis, gastroenteritis, allergic purpura, epilepsy, Asthma attack, Convulsion, Myocarditis.

## 4 Discussion

Respiratory infections are the most common illness during childhood, primarily caused by a diverse array of pathogens. With more than 200 identified serotypes, early screening for specific etiological agents is critical for guiding clinical diagnosis and treatment. The six pathogens investigated in this study are among the most prevalent causes of pediatric respiratory infections in recent years (19–21). In our cohort, 34,505 cases tested positive for at least one pathogen, yielding an overall positivity rate of 53.50%, which was higher than those reported in other studies (22, 23). The co-infection rate in the present study was 7.55%, also higher than the 2.5% documented by Zeng et al. (23). Among the six pathogens, HRV exhibited the highest detection rate (18.67%), followed by MP at 14.93%. In contrast, Zhang et al. (21) reported a much lower HRV positivity rate (6.19%) and an even lower MP rate (2.43%) in a study of children with influenza-like illness between 2020 and 2021. The higher detection rates of HRV and MP observed in our study relative to previous reports (24, 25) may be attributed to differences in study periods, geographic regions, and laboratory detection methods (26–28). Previous investigations have indicated that MP epidemics exhibit cyclic surges every 3–7 years, potentially driven by antigenic variation or waning herd immunity (29).

In this study, we found that the overall infection rate was higher in males than in females, which is consistent with previous studies (22, 30). This might be explained by boys have more alveoli than females but the same mean alveolar size; therefore, they have larger lungs at any given age. This anatomical difference may increase the opportunities for pathogens to colonize and cause infections in the respiratory tract of males (31). Boys tend to have broader activity ranges and a greater preference for outdoor sports, which may increase their exposure to pathogens. However, susceptibility to different pathogens varies between sexes. For example, the positivity rates for MP and FluB were higher in females than in males, consistent previous studies (23). In contrast, the positive rate of HRV was significantly higher in males than in females. No significant differences were observed in the positivity rate of RSV, FluA and ADV between sexes. Previous literature also reported that no significant sex differences in common respiratory viral infections including RSV (26), while some studies reported higher detection rate in males than in females (31, 32). These discrepancies may be associated with climatic and environmental factors, population distribution, economic status and diagnostic methods used.

We also found that different pathogens exhibit varying susceptibility among children of different ages: RSV predominantly infects young children, with a high infection rate among those aged 0–3 years that gradually declines to below 10% by age 4. This is possibly because RSV is a single-stranded RNA virus that is more likely to infect younger children (33). In contrast, the infection rates of MP and ADV were low among infants under 1 year of age but increased significantly after 1 year of age. This is mainly because MP and ADV are transmitted via airborne droplets and can also cause infection through contact with infected children or carriers, rendering older children more susceptible. The infection trends for FluB and FluA remained relatively flat without distinct peaks. Vaccination is likely a key factor, as it helps reduce the overall number of infections and limits the spread of these viruses (34). HRV exhibited a high infection rate across all age groups of children, with a disproportionately higher prevalence in those younger than 6 years, which may be explained by the increased susceptibility of younger children to viral infection compared with older children.

The overall positivity rates of the six respiratory pathogens were highest in winter (59.09%) and lowest in summer (47.49%), which is consistent with previous findings (13). This phenomenon may be explained by the fact that cold, dry air provides a stable environment for viral survival, while low temperatures lead to increased crowding in poorly ventilated indoor spaces, thereby facilitating viral transmission. Positive rates for RSV, FluA, and FluB peaked in winter. In contrast, MP and HRV showed the highest detection rates mainly in autumn. Although previous studies have suggested that ADV infection exhibits no obvious seasonality (35, 36), the highest ADV positive rate in this study occurred predominantly in summer. This observation is consistent with the report by Botti (37,38), who noted that ADV is mainly prevalent in summer. This pattern may be attributed to the fact that ADV can be transmitted not only via respiratory droplets but also through the fecal–oral route and contaminated water sources.

From July 2023 to January 2024, MP was the most common pathogenic agent among the six pathogens, with the exception of September 2023. The positive rate of MP exceeded 20% in July 2023, then increased significantly, peaking in October 2023. Thus, this period (July 2023 to March 2024) may have corresponded to a large-scale MP infection outbreak in Chengdu.

The positive rate of ADV increased significantly in April 2024, peaking in July 2024 before falling. However, as of December 2024(15.20%), the positive rate of ADV remained above 10%. This may be due to annual variations in the circulation pattern of ADV or an ADV infection outbreak in 2024. HRV was prevalent throughout the year, which is consistent with previous reports (23). So such year-to-year variations in the epidemiological patterns of respiratory infections confirm the importance of long-term epidemiology studies.

Pneumonia was the predominant disease type caused by the six respiratory pathogens, possibly because the study populatio consisted mostly of hospitalized patients with more severe conditions, and these pathogens can also cause severe pneumonia. Among pneumonia patients, the <1-year-old group had the highest positive rate across all age groups, except for MP. This may be attributed to the weak immune function of infants: their living range is relatively confined, mainly limited to the household, they interact with a fixed group of individuals, and have less exposure to MP. In addition, the six respiratory pathogens may also be associated with causing herpangina, gastroenteritis, acute wheezing attack, myocardial damage, infectious mononucleosis, Henoch-Schonlein purpura and other diseases, which should also be given more attention in clinical practice.

### 4.1 Limitations

(1) This study is a single-center study with a limited study population, Given the certain regional differences in respiratory pathogen infections, the present study only reflects pathogens infections in Chengdu. (2) Due to objective reasons, we did not perform detection tests for the six pathogens before September 2022, so we cannot conduct comparative statistics between the COVID-19 pandemic and the post-pandemic period. (3) For hospitalized cases, routine blood culture testing was not performed. Consequently, this study cannot assess the incidence of concurrent bloodstream infections in children with respiratory pathogen infections which constrains a more comprehensive understanding of disease severity and co-infection patterns. (4) This study was limited to six common pathogens and did not include bacterial culture or a more comprehensive panel of viral agents. This constraint may have resulted in an underestimation of the true etiology of respiratory infections in the study population. (5) There was an imbalance in the proportion of inpatients and outpatients across different age groups, which may introduce potential bias into this study. In general, compared with outpatients, inpatients tend to have more severe clinical manifestations and are more likely to undergo comprehensive pathogen detection. In this study, the higher hospitalization rate in children aged <1 year may lead to an overestimation of the positivity rate and disease severity in this age group. Therefore, in future studies, we will stratify the study population by inpatient/outpatient status and adjust for this imbalance in statistical analysis to reduce such bias, thereby improving the reliability and generalizability of the study results. (6) Data on vaccination status, particularly for influenza, were not available. The absence of this information may have confounded the interpretation of FluA and FluB positivity rates and severity.

## 5 Conclusion

Among the six respiratory pathogens, HRV and MP became the main pathogens of respiratory tract infections in children in Chengdu from September 2022 to December 2024. Flu A and FluB were associated with lower-level infection. In addition, different respiratory pathogens exhibited different epidemic characteristics across different age groups, sexes, and seasons. Therefore, in clinical practice, it is necessary to closely monitor the epidemiological trends of these pathogens.

## Data Availability

The original contributions presented in the study are included in the article/Supplementary Material, further inquiries can be directed to the corresponding author.
